# Successful treatment of PLA_2_R1-antibody positive membranous nephropathy with ocrelizumab

**DOI:** 10.1007/s40620-020-00874-2

**Published:** 2020-10-07

**Authors:** Tilman Schmidt, Matthias Schulze, Sigrid Harendza, Elion Hoxha

**Affiliations:** 1 III. Department of Internal Medicine, University Medical Center Hamburg-Eppendorf, Martinistr. 52, 20246 Hamburg, Germany; 2 Viamedis Renal Center Bad Zwischenahn, Bad Zwischenahn, Germany

**Keywords:** Membranous nephropathy, Rituximab, Ocrelizumab, Nephrotic syndrome

## Abstract

Membranous nephropathy (MN) is a common cause of nephrotic syndrome in adults. The disease is induced by antibodies, which are directed against the podocyte protein phospholipase A_2_ receptor 1 (PLA_2_R1-ab) in 80% of cases. B cell depleting treatments, most notably rituximab, a chimeric CD20-antibody, are often effective for treatment of MN. However, in 35–40% of patients rituximab fails to induce remission of disease and relapses after rituximab-induced remission are frequent. Therefore, alternative treatment options are necessary. Over the past years optimized antibodies targeting CD20 were designed to overcome side effects or sensitization to the murine fractions of rituximab and potentially improve B cell depletion. Ocrelizumab is a humanized B cell depleting antibody, approved for treatment of multiple sclerosis (MS). Here, we report the case of a patient who was diagnosed with MS and, 8 years later, developed PLA_2_R1-associated MN. Treatment for MS was switched to the CD20-antibody ocrelizumab, which was expected to deplete B cells and potentially induce remission of MN. After treatment with ocrelizumab PLA_2_R1-ab disappeared from the circulation and the patient developed remission of proteinuria. Ocrelizumab might be an efficacious treatment alternative for patients with MN who fail to achieve remission or are immunologically sensitized to rituximab.

**Electronic supplementary material:**

The online version of this article (10.1007/s40620-020-00874-2) contains supplementary material, which is available to authorized users.

## Introduction

Membranous nephropathy (MN) is an autoimmune disease and a common cause of nephrotic syndrome in adults. MN has been associated with different disease conditions, e.g. chronic infections (hepatitis B), malignancies, or different autoimmune diseases in up to 25% of cases, which are then considered secondary [1]. Phospholipase A_2_ receptor 1 (PLA_2_R1) is the major target antigen in MN and up to 80% of patients have circulating antibodies directed against this protein [2]. Autoantibody binding leads to formation of immune deposits on the glomerular basement membrane, ultimately causing nephrotic syndrome due to loss of proteins in the urine. In the long term, about one-third of patients will achieve spontaneous remission of proteinuria, especially patients with low PLA_2_R1-antibody level [3–5]. At the same time, approximately 16% of patients with MN develop end-stage renal disease over 5–10 years [3]. Detection of PLA_2_R1-antibody (PLA_2_R1-ab) in the blood and staining of PLA_2_R1 in the renal biopsy allow diagnosis of MN [6, 7]. Moreover, PLA_2_R1-ab levels predict long-term renal outcome and their reduction precedes spontaneous or immunosuppressive-induced clinical remission [1, 4, 8]. Therefore, repetitive measuring of PLA_2_R1-ab levels is a powerful tool to guide therapy. If immunosuppressive therapy is indicated, different drugs are available, e.g. cyclosporine A, alkylating agents, or rituximab. The MENTOR study showed that rituximab was superior to cyclosporine A for achieving remission of proteinuria after 24 months [9]. Although randomized controlled studies are still lacking, a number of publications shows that cyclophosphamide is inferior to rituximab regarding serious and non-serious adverse events [10]. The chimeric CD20 antibody rituximab has shown positive results for treatment of MN [11]. At the same time, several studies have shown that in up to 35–40% of cases rituximab does not induce remission of disease. Relapses appear in about 25% of patients with PLA_2_R1-associated MN, making repeated treatment cycles necessary [11, 12]. In such cases, immunological sensitization to the murine parts of rituximab has been reported. In contrast to rituximab, ocrelizumab is a humanized antibody targeting CD20. It has been suggested that it might allow a more effective B cell depletion by antibody-dependent cell-mediated cytotoxicity [13]. Ocrelizumab is indicated for treatment of multiple sclerosis (MS) and is the first approved drug for primary progressive MS [14]. MS is typically a relapsing–remitting demyelinating autoimmune disease of the central nervous system (CNS). Formation of autoreactive B cell clones is suspected to play a major pathogenic role in MS [15]. In this case report, we show for the first time that ocrelizumab is effective for the treatment of PLA_2_R1-associated MN, inducing a clinical and serological remission.

## Case report

**Fig. 1:**
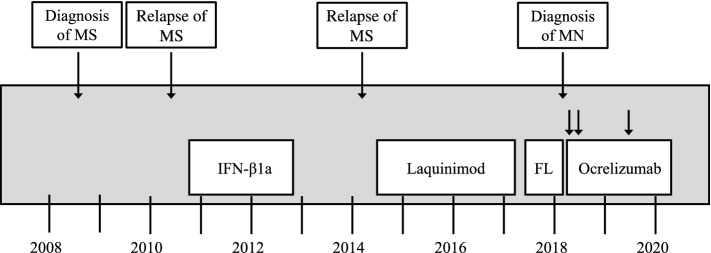
Course of diseases and treatment. Multiple sclerosis (MS) was diagnosed in June 2008 and was treated with interferon-beta1a (IFN-β1a) since October 2010. Due to disease progression of MS, therapy was switched to laquinimod and, 3 years later, to fingolimod (FL). Membranous nephropathy (MN) was diagnosed in January 2018 and treatment with ocrelizumab was started 6 months later

**Fig. 2:**
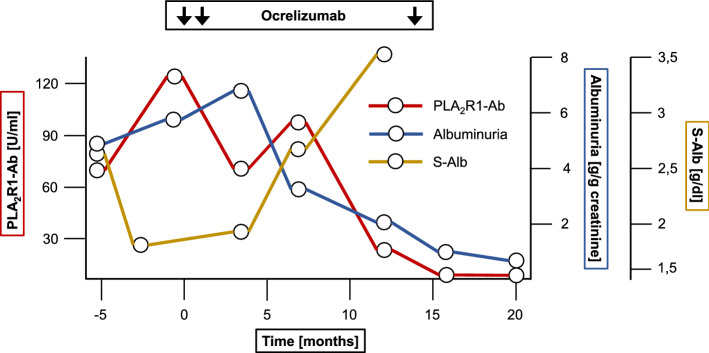
PLA_2_R1-ab, albuminuria and serum albumin during treatment with ocrelizumab. *S-Alb* serum albumin, *PLA*_*2*_*R1-ab* PLA_2_R1-antibody

A 52-year-old male Caucasian patient was referred to our outpatient clinic with nephrotic-range proteinuria. PLA_2_R1-ab level was 68 U/ml and renal biopsy confirmed the diagnosis of PLA_2_R1-associated MN. Eight years earlier, the patient had been diagnosed with MS. He was first treated with beta-1a interferons for 2 years and laquinimod for 3 years but showed progression of MS under both therapeutic strategies. Therefore, treatment was changed to fingolimod, upon which the patient showed no further disease progression for 10 months until 2018, when MN was diagnosed (Fig. 1). The patient initially presented with progressive edema, hypertension, nephrotic syndrome (albuminuria 5.15 g/day, serum albumin 2.7 g/dl, serum cholesterol 326 mg/dl), and a serum creatinine of 1.11 mg/dl. After an initial supportive treatment strategy with an ACE-inhibitor and diuretics for 6 months, the patient demonstrated disease progression with albuminuria increasing to 6.02 g/day and serum creatinine of 1.84 mg/dl. PLA_2_R1-ab also increased to 123 U/ml (Fig. 2). Due to the ongoing immunosuppressive medication for MS, a therapeutic approach had to be chosen to treat both diseases without over-suppressing the immune system of the patient. Therefore, we refrained from adding a second immunosuppressive treatment for MN (e.g. cyclophosphamide, calcineurin inhibitors or rituximab) in addition to the current treatment for MS. Rather, a B cell depleting therapy with ocrelizumab, which is proven to be effective for MS treatment, was chosen, assuming the CD20-antibody would be effective in additionally treating MN. The patient received two doses of ocrelizumab (300 mg each) within 2 weeks. After 16 weeks B cells were depleted to 3 B cells/µl (normal range 80–500 B cells/µl). After 12 months, PLA_2_R1-ab had declined to 21 U/ml (threshold of the ELISA-test for PLA_2_R1-ab positivity is 20 U/ml) [16]. At this time, B cells remained low at 3 cells/µl, the patient had a partial remission of albuminuria (2.32 g/day) and serum albumin had increased to 3.5 g/dl. After 14 months, the patient received one additional dose of ocrelizumab (300 mg) because of clinical progression of MS. Again, treatment with ocrelizumab lead to depletion of B cells (0 cells/µl 20 weeks after infusion of ocrelizumab). Twenty months after initiation of treatment, PLA_2_R1-ab had completely disappeared from the circulation (PLA_2_R1-ab 2 U/ml) and albuminuria was lowered to 0.95 g/day. Currently, 2 years after treatment with ocrelizumab, no relapses have occurred, neither of MS nor MN, and PLA_2_R1-ab remain negative.

## Discussion

A relevant number of patients with MN show a progressive deterioration of renal function or develop end stage renal disease (ESRD) [3]. Since MN is an antibody-induced disease and PLA_2_R1-ab are identified in 80% of patients, B cell depletion with rituximab has been increasingly used in patients with MN. Rituximab was shown to be safe and efficacious in inducing remission of proteinuria but, failed to induce remission of proteinuria in 40% of patients [9, 11]. Moreover, approximately 28% of patients developed an allergic reaction upon infusion of rituximab, requiring in some cases treatment with high dose steroids. It has been shown that antibodies against rituximab are frequently found during the course of therapy and are associated with a faster reconstitution of B cells, higher proteinuria, and can cause serum sickness after repetitive rituximab infusions [12]. To overcome immunogenicity of rituximab and improve B cell depletion, other CD20-depleting treatment strategies have been designed (Supplement Table 1). For rituximab several mechanisms have been suggested to mediate cytotoxicity on B cells. These include antibody-dependent cellular toxicity (ADCC) that is regulated by Fc receptors on effector cells (e.g. NK cells, macrophages and dendritic cells) [17, 18]. Complement-dependent cytotoxicity (CDC) involves fixation of complement factors and subsequently lysis of targeted cells [19, 20]. In contrast to rituximab, which is a chimeric antibody, ocrelizumab is a humanized antibody and both drugs target different epitopes of CD20 [21]. Rituximab consists of a non-human variable region and a human constant region, while ocrelizumab comprises only a small non-human hypervariable region. The mechanism of action is also different between ocrelizumab and rituximab. Depletion of antibody-targeted cells is mediated by the complement system and/or cellular toxicity. In vitro data suggest, that ocrelizumab’s mechanism of B cell depletion relies more on antibody-dependent cellular toxicity (ADCC) and less on complement-dependent cytotoxicity (CDC), compared to rituximab [13, 21]. Ocrelizumab is approved for treatment of relapsing–remitting and primary progressive MS and it was associated with lower rates of disease activity when compared to interferon beta-1a [12].

In this case report, ocrelizumab was used in a 52-year-old male patient who suffered from MS, which was diagnosed 8 years prior to the diagnosis of MN. To avoid double immunosuppression with alkylating drugs, calcineurin inhibitors, or rituximab, in addition to MS treatment, we chose a therapy strategy targeting both diseases, MS and MN. With ocrelizumab we had the opportunity to treat MS with an approved immunosuppressive drug, while simultaneously successfully targeting PLA_2_R1-ab producing B cells and thus curing MN. Ocrelizumab effectively depleted B cells with consecutive PLA_2_R1-ab depletion after 6–12 months. Considering the fact that PLA_2_R1-ab levels strongly correlated with the clinical outcome in patients with MN, measurement of autoantibody levels is important to judge treatment efficacy [8]. At the same time, measurement of B cells is helpful in cases when PLA_2_R1-ab do not deplete, to decide whether an alternative B cell depleting agent might be indicated.

In summary, ocrelizumab should be considered for therapy of MN if rituximab is not feasible, i.e. because of inefficacy, allergic reaction, or immune sensitization to the drug. With this case report, we provide evidence for the first time that ocrelizumab, a humanized CD20 antibody, is effective in treating PLA_2_R1-ab positive MN. Treatments with alternative CD20-antibodies might be helpful for the 35–40% of patients with MN, in whom rituximab treatment fails.

## Electronic supplementary material

Below is the link to the electronic supplementary material.

Supplementary material 1 (DOCX 28 kb)
