# Supplementary material for: Successful treatment of PLA2R1-antibody positive membranous nephropathy with ocrelizumab
Source: J Nephrol. 2020 Oct 7;34(2):603–6. doi: 10.1007/s40620-020-00874-2 (PMC8036191; doi:10.1007/s40620-020-00874-2)
Supplement: Supplementary file 1 — Supplementary material 1 (DOCX 28 kb) [file 40620_2020_874_MOESM1_ESM.docx]

| **Drug** | **Structure** | **Mechanism of action** | **Depleting effect** | **Indications**  **(FDA approved)** |
| --- | --- | --- | --- | --- |
| **Rituximab** | Chimeric | - Type I Ab | - CDC [1, 2] - ADCC [3, 4] - direct induction of apoptosis [5, 6] | NHL, CLL, RA, GPA, MPA, PV |
| **Ocrelizumab** | Humanized | - Type I Ab - Overlapping epitope binding compared to Rituximab [7, 8] | - Enhanced ADCC and less CDC compared to Ritximab [9] | RRMS, PPMS |
| **Veltuzumab** | Humanized | - Type I Ab - Similar antigen binding to Rituximab [10] - Enhanced binding avidity compared to Rituximab [10] | - Stronger CDC [11] | Orphan Drug  (ITP) |
| **Obinutuzumab** | Humanized | - Type II Ab - Partial overlap with Rituximab [12] | - Enhanced ADCC - Less CDC [12] | CLL,  FL |
| **Ofatumumab** | Fully human | - Type I Ab - Binds to a different epitope than Rituximab [13] | - Improved CDC and ADCC compared to Rituximab [14] | CLL |

Supplement Table 1: FDA approved CD20-antibodies

Abbr.: CDC – complement-dependent cytotoxicity; ADCC – antibody-dependent cellular cytotoxicity; NHL – non-Hodgkin-lymphoma; CLL – chronic lymphatic leukemia; RA – rheumatoid arthritis; GPA – granulomatosis with polyangiitis; MPA – microscopic polyangiitis; PV – pemphigus vulgaris; RRMS – remitting-relapsing multiple sclerosis; PPMS – primary progressive multiple sclerosis; ITP – idiopathic thrombopenic purpura; FL – follicular lymphoma

References

1. Golay J, Zaffaroni L, Vaccari T, et al (2000) Biologic response of B lymphoma cells to anti-CD20 monoclonal antibody rituximab in vitro: CD55 and CD59 regulate complement-mediated cell lysis. Blood 95:3900–3908

2. Zhou X, Hu W, Qin X (2008) The Role of Complement in the Mechanism of Action of Rituximab for B‐Cell Lymphoma: Implications for Therapy. The Oncologist 13:954–966. https://doi.org/10.1634/theoncologist.2008-0089

3. Manshouri T, Do K, Wang X, et al (2003) Circulating CD20 is detectable in the plasma of patients with chronic lymphocytic leukemia and is of prognostic significance. Blood 101:2507–2513. https://doi.org/10.1182/blood-2002-06-1639

4. Lefebvre M-L, Krause SW, Salcedo M, Nardin A (2006) Ex Vivo-activated Human Macrophages Kill Chronic Lymphocytic Leukemia Cells in the Presence of Rituximab: Mechanism of Antibody-dependent Cellular Cytotoxicity and Impact of Human Serum: J Immunother 29:388–397. https://doi.org/10.1097/01.cji.0000203081.43235.d7

5. Byrd JC, Kitada S, Flinn IW, et al (2002) The mechanism of tumor cell clearance by rituximab in vivo in patients with B-cell chronic lymphocytic leukemia: evidence of caspase activation and apoptosis induction. Blood 99:1038–1043. https://doi.org/10.1182/blood.V99.3.1038

6. Pedersen IM, Buhl AM, Klausen P, et al (2002) The chimeric anti-CD20 antibody rituximab induces apoptosis in B-cell chronic lymphocytic leukemia cells through a p38 mitogen activated protein–kinase–dependent mechanism. Blood 99:1314–1319. https://doi.org/10.1182/blood.V99.4.1314

7. Martin P, Furman RR, Ruan J, et al (2008) Novel and Engineered Anti–B-Cell Monoclonal Antibodies for Non-Hodgkin’s Lymphoma. Semin Hematol 45:126–132. https://doi.org/10.1053/j.seminhematol.2008.02.007

8. Robak T (2008) Novel Monoclonal Antibodies for the Treatment of Chronic Lymphocytic Leukemia. Curr Cancer Drug Targets 8:156–171. https://doi.org/10.2174/156800908783769319

9. Kausar F, Mustafa K, Sweis G, et al (2009) Ocrelizumab: a step forward in the evolution of B-cell therapy. Expert Opin Biol Ther 9:889–895. https://doi.org/10.1517/14712590903018837

10. Goldenberg DM, Rossi EA, Stein R, et al (2009) Properties and structure-function relationships of veltuzumab (hA20), a humanized anti-CD20 monoclonal antibody. Blood 113:1062–1070. https://doi.org/10.1182/blood-2008-07-168146

11. Goldenberg DM, Morschhauser F, Wegener WA (2010) Veltuzumab (humanized anti-CD20 monoclonal antibody): characterization, current clinical results, and future prospects. Leuk Lymphoma 51:747–755. https://doi.org/10.3109/10428191003672123

12. Bologna L, Gotti E, Manganini M, et al (2011) Mechanism of Action of Type II, Glycoengineered, Anti-CD20 Monoclonal Antibody GA101 in B-Chronic Lymphocytic Leukemia Whole Blood Assays in Comparison with Rituximab and Alemtuzumab. J Immunol 186:3762–3769. https://doi.org/10.4049/jimmunol.1000303

13. Teeling JL, Mackus WJM, Wiegman LJJM, et al (2006) The Biological Activity of Human CD20 Monoclonal Antibodies Is Linked to Unique Epitopes on CD20. J Immunol 177:362–371. https://doi.org/10.4049/jimmunol.177.1.362

14. Cheson BD (2010) Ofatumumab, a Novel Anti-CD20 Monoclonal Antibody for the Treatment of B-Cell Malignancies. J Clin Oncol 28:3525–3530. https://doi.org/10.1200/JCO.2010.27.9836
